# Heat Stress Response in *Ruditapes Decussatus*: Transcriptional Regulation of Key Pathways and Novel SNPs in Antioxidant Genes

**DOI:** 10.1002/ece3.71563

**Published:** 2025-06-10

**Authors:** Dimitrios K. Papadopoulos, Basile Michaelidis, Ioannis A. Giantsis

**Affiliations:** ^1^ Laboratory of Animal Physiology, Department of Zoology, Faculty of Science, School of Biology Aristotle University of Thessaloniki Thessaloniki Greece; ^2^ Laboratory of Ichthyology & Fisheries, Faculty of Agriculture, Forestry and Natural Environment Aristotle University of Thessaloniki Thessaloniki Greece

**Keywords:** antioxidant defense, apoptosis, climate change, genetic markers, metabolic regulation, selection

## Abstract

Global warming significantly impacts coastal zones, particularly affecting ectothermic inhabitants such as bivalve mollusks. This study evaluates the response of the grooved carpet shell clam *Ruditapes decussatus* (Linnaeus, 1758) to increasing temperatures (22.5°C, 24.5°C, 26.5°C) over 25 days through the transcription of key genes involved in antioxidant defense [*Cu‐Zn superoxide dismutase (Cu‐Zn sod)*, *catalase*, *metallothionein*], anti‐apoptotic procedures [*b‐cell lymphoma 2 (bcl2*)], and energy metabolism [*pyruvate kinase (pk)*, *phosphoenolpyruvate carboxykinase (pepck*)]. Additionally, the genes *catalase* and *Cu‐Zn sod* were characterized for the first time, and along with the *metallothionein* gene, were sequenced in heat‐resilient and heat‐susceptible individuals to identify polymorphisms potentially associated with thermal tolerance. At 22.5°C, clams showed a delayed increase in glycolytic flux and a gradual up‐regulation of antioxidant and anti‐apoptotic mechanisms. At 24.5°C and 26.5°C, a strong initial stress response resulted in equally high mortality during the early days of exposure. Subsequently, clams appeared to shift toward a reduced energy metabolism, with mildly upregulated antioxidant defenses and anti‐apoptotic activity. With prolonged exposure, there was evidence of enhanced aerobic glycolysis, antioxidant, and anti‐apoptotic responses at these temperatures. However, increased *pepck* transcription on days 12 and 25 suggests that metabolic demands may have exceeded aerobic capacity, potentially triggering apoptotic processes at 26.5°C. Clams at 24.5°C maintained aerobic capacity upon the final day, also engaging anaerobic pathways to meet energy demands. Eventually, three SNPs were statistically correlated with heat resilience. These included one non‐synonymous SNP in *catalase*, one SNP in the 3′ untranslated region (3′UTR) of *metallothionein*, and one synonymous SNP in *Cu‐Zn sod*. These findings underscore the sensitivity of *R. decussatus* populations from the northeastern Mediterranean to persistent thermal stress and reveal several polymorphisms in antioxidant genes with potential adaptive significance. However, the limited sample size and the weak correlations observed in some cases highlight the need for further research to clarify the relationship between these polymorphisms and thermal resilience.

## Graphical abstract

Climate change is known to affect all levels of biology, setting at risk ecological balance. Among other organisms' groups, aquatic inhabitants, and particularly bivalves, are expected to be influenced to a greater extent owing to their inability to move to more mild temperatures. Therefore, these organisms represent excellent models to study the adaptation to global warming. Keeping this in mind, in the present study, we molecularly characterized the evolutionary response of the marine bivalve Ruditapes decussatus to increased seawater temperature by means of both antioxidant gene expression and resilience‐associated genetic loci. Several genes were characterized for the first time, and novel SNPs were detected that are linked with tolerance to climate change.

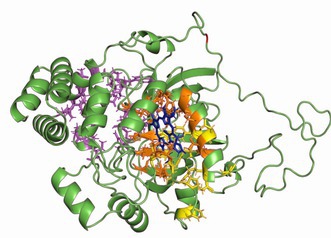

## 1 Introduction

The grooved carpet shell clam *Ruditapes decussatus* is a burrowing bivalve typically inhabiting sandy and silty mud substrates in the mid‐intertidal zone at depths ranging from 15 to 20 cm to a few meters (FAO 2021). It is widely distributed along the European, Mediterranean, and Northern African coasts, lagoons, and estuaries (Matias et al. 2009). Its ecological role as a filter feeder contributes to nutrient cycling and sediment stability, making it a key species in coastal ecosystems. The species exhibits a gonochoric reproductive system, with external fertilization that occurs mainly during the summer months. Brood size can vary significantly depending on parental condition and environmental factors (Ojea et al. 2008). Embryos develop into free‐swimming trochophore larvae, and later into veliger larvae before settling into benthic habitats.

*R. decussatus* is of great commercial value in Europe (FAO 2022), with significant and unfulfilled consumer demand (Cruz et al. 2020). Populations of this species also inhabit Greek coasts, representing a high‐value fishery product (Papadopoulos et al. 2024). Nevertheless, the Manila clam, *Ruditapes phillipinarum*, dominates the global clam aquaculture yields due to its faster growth and adaptability to different environments (Coelho et al. 2021). Following the introduction of the congener alien species, the captures of *R. decussatus* in its native range have been reduced substantially (FAO, 2021). *R. philippinarum* has higher fecundity (Rato et al. 2022) and shows greater resilience to environmental stressors, outperforming the native clam under heat stress conditions (Crespo et al. 2021). Recently, climate change‐induced mass mortality events have led to significant population declines of *R. decussatus* in Northern Spain (Mihara and Donback 2024). The high market value of *R. decussatus* makes its cultivation potentially more attractive than that of *R. philippinarum*. In Portugal, *R. decussatus* is vital to the revenue of the aquaculture industry (De Marchi et al. 2020). The aquaculture production of the grooved carpet shell clam could be expanded by suspension culture techniques, where similar or better growth and survival have been recently evidenced in experimental field trials (Anhichem et al. 2021; Medlouh et al. 2023). Considering its commercial importance and the increasing threats from environmental stressors, especially those driven by climate change, it is essential to investigate the stress response mechanisms of *R. decussatus* to support sustainable management and aquaculture development.

Global mean sea surface temperature has increased from the beginning of the 20th century by about 0.88°C (Cooley et al. 2022). Until 2100, an additional increase of 0.86°C–2.89°C is estimated to occur based on climate model projections (Fox‐Kemper et al. 2021). Coastal zones could be especially affected by warming through decreased breeding places, loss of aquatic biodiversity, and formation of extended hypoxic zones (Eissa and Zaki 2011). Estuaries, for instance, are heated faster than many other ecosystems (MacKenzie and Schiedek 2007). Global warming has increased the marine heatwaves, which represent periods of extremely elevated seawater temperature that last for days to months, commonly generating severe problems for aquatic ecosystems and their services (Smale et al. 2019). Heat waves have become more frequent, intense, and persistent since the beginning of the 21st century (Fox‐Kemper et al. 2021). Due to these episodes, several aquatic species face conditions beyond their tolerance limits (Cooley et al. 2022). Ectotherms, including bivalve mollusks, are particularly affected because their metabolism depends directly on oxygen supply, which decreases at high temperatures (Pörtner 2001). Thermal stress, driven by climate change, can disrupt physiological homeostasis in marine bivalves, leading to oxidative damage, impaired cellular survival, and metabolic imbalance. Therefore, our study focused on the transcriptional regulation of genes that represent critical pathways determining thermal resilience in bivalves.

Reactive oxygen species (ROS) in marine bivalves raise following exposure to heat stress (Rahman et al. 2019; Wang et al. 2018; Yin et al. 2017). Under normal conditions, there is a balance between the formation and elimination of ROS, which is attributed to the effective removal of ROS by the antioxidant defense systems. The antioxidant machinery contains enzymatic (catalase, superoxide dismutase, glutathione peroxidase) and non‐enzymatic (ascorbic acid, glutathione, metallothionein) components, which cooperate to eliminate the surplus of produced ROS (Sies 1993). Under stressful conditions, rapid removal of excessive ROS is essential to maintain cellular homeostasis. Bivalves respond to the increased ROS generated during heat stress by activating their antioxidant defense mechanisms. Among these, the enzyme superoxide dismutase (SOD, EC 1.15.1.1) catalyzes the dismutation of the superoxide anion (O_2_^•−^) into molecular oxygen (O_2_) and hydrogen peroxide (H_2_O_2_). This reaction is critical for maintaining cellular redox balance under stress conditions. Additionally, catalase (CAT, EC 1.11.1.6) further detoxifies hydrogen peroxide by decomposing it into water and oxygen. The activation of these enzymes is a key aspect of the cellular response of bivalves to thermal stress (Buttemer et al. 2010). Metallothioneins (MTs) are a superfamily of cysteine‐rich, low molecular weight, and highly conserved proteins. The activity of MTs in scavenging hydroxyl radicals and their inhibition activity against microsomal lipid peroxidation was found to be 50 and 10 times higher, respectively, than the activity of reduced glutathione (Miura et al. 1997). The important antioxidant properties of MTs have been characterized many years back in bivalves (Anderson et al. 1999; Buico et al. 2008).

When the antioxidant machinery is overwhelmed, cellular damage occurs, and apoptosis may be stimulated (Abele and Puntarulo 2004). The activation of apoptotic procedures following oxidative damage has been evidenced in bivalves (Tang et al. 2022; Zhang, Yan, et al. 2020; Zhang, Storey, et al. 2020). Apoptosis is a highly conserved mechanism of multicellular organisms, where the induction of programmed cell death contributes to homeostasis. Two main distinct pathways are involved in apoptosis: the death receptor‐mediated (extrinsic) pathway and the mitochondrial (intrinsic) pathway. The B cell lymphoma 2 (BCL‐2) protein family comprises several conserved proteins that are key regulators of the intrinsic apoptotic pathway (Chipuk et al. 2010). Bcl‐2 Associated X‐protein (BAX) is involved in the pro‐apoptotic action by regulating autophagy and thus represents an important indicator for cell death responses (Bermejo‐Nogales et al. 2014). The well‐known anti‐apoptotic BCL‐2 protein inhibits the cell death signaling cascade by preventing the release of cytochrome c from mitochondria and advocates cell survival through the suppression of the death‐promoting activity of BAX (Chipuk et al. 2010).

The thermal vulnerability of ectotherms is closely determined by the effects of elevated temperature on cellular bioenergetics and especially on mitochondrial functions (Hunter‐Manseau et al. 2019). Metabolic genes can be implemented as useful indicators of adaptive responses to temperature variations in marine organisms (Kenkel et al. 2013). Enzymes implicated in mitochondrial energy production, such as phosphoenolpyruvate carboxykinase (PEPCK) and pyruvate kinase (PK), play crucial roles in these metabolic adaptations (Bermejo‐Nogales et al. 2014; Morley et al. 2009). PK is a key enzyme for the aerobic production of ATP through glycolysis, while PEPCK participates in metabolic pathways by regulating gluconeogenesis and represents an important marker of metabolic stress (Bermejo‐Nogales et al. 2014).

Warming sharpens coastal eutrophication and associated hypoxia, forming oxygen‐depleted areas, which are detrimental to coastal ecosystems and lead to mass mortalities, habitat depletion, and fisheries disturbances (Cooley et al. 2022). Elevated temperatures commonly lead to an increase in the metabolic rates in bivalves to ensure sufficient energy supply (Sokolova et al. 2012; Solan and Whiteley 2016). However, when body temperature approaches a critical threshold for survival, metabolic depression arises to conserve energy (Storey and Storey 1990). In addition, the efficiency of ATP production at higher temperatures is reduced due to imbalances in oxygen supply and needs, which lead to a transition to anaerobic metabolism impacting physiological performance (Zeis et al. 2023). A crucial adaptive strategy for surviving reduced oxygen availability involves suppression of metabolic rate and restriction of macromolecule biosynthesis (Storey and Storey 2004). Under hypoxia, except for the ability to sustain a decreased metabolism, bivalves activate special anaerobic glycolysis with the participation of PEPCK that increases the ATP output and contributes crucially to hypoxia tolerance (Larade and Storey 2009). Aquatic organisms exhibit resilience to temperature fluctuations within a certain tolerance range (Somero 2002). Historically, different species have managed to cope with climate shifts over their evolutionary timelines. However, the rapid pace of current climate change may challenge their ability to adapt (Philippart et al. 2003). The acclimation to changing environments is attributed to physiological and behavioral adaptations from the different species to the variable marine environment. These adjustments may have an underlying genetic background.

In this study, we investigated the transcriptional regulation of key stress‐responsive genes in the clam *R. decussatus*, during a 25‐day exposure to progressively elevated ambient temperatures (22.5°C, 24.5°C, and 26.5°C), aiming to assess the potential impact of sustained thermal stress conditions that are increasingly frequent and intense due to climate change. In contrast to tidal flat environments where *R. decussatus* may experience extreme temperature fluctuations exceeding 30°C during tides (Macho et al. 2016), the clams used in this study inhabit shallow waters that are not subject to tidal exposure. In Vistonikos Bay, the average sea surface temperature during August, which is the warmest month, typically ranges between 26°C and 28°C (SeaTemperature.info 2024). Considering that mobile bivalve species usually employ behavioral thermoregulation strategies to avoid excessive heat, such as moving to lower shore areas or burrowing to greater depths (Zhang, Yan, et al. 2020; Zhang, Storey, et al. 2020), the highest temperature in this study was selected to approximate the peak temperature that *R. decussatus* experiences during summer.

The transcription of key genes involved in antioxidant defense (*Cu‐Zn superoxide dismutase*, *catalase*, *metallothionein*), anti‐apoptotic processes (*bcl2*), and metabolism (*pyruvate kinase*, *phosphoenolpyruvate carboxykinase*) was studied at the different temperatures for 25 days. Since the expression of the aforementioned genes has not been studied in *R. decussatus*, multiple novel primers were designed. Additionally, the sequences of the three antioxidant genes were analyzed in heat‐resilient and heat‐susceptible animals to identify polymorphic loci potentially associated with thermal stress tolerance. The goal of genotyping individuals with distinct thermal stress tolerance phenotypes was to identify potential markers for selective breeding, which could enhance aquaculture practices and promote the sustainability of wild *R. decussatus* populations in the face of global warming.

## 2 Materials and Methods

### 2.1 Animal Acclimation and Daily Routine

**FIGURE 1:**
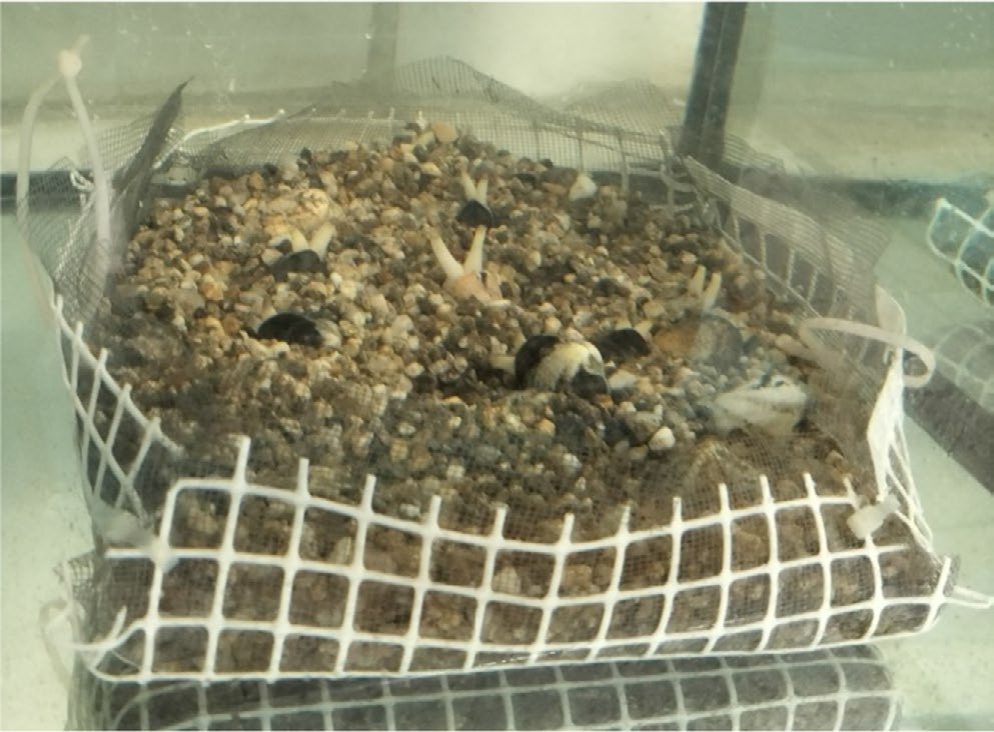
The baskets where *Ruditapes decussatus* clams were placed for the experiment.

Around 600 *R. decussatus* adult clams (20.12 ± 1.79 g wet weight, 4.09 ± 0.38 cm shell length) were collected from wild populations in Vistonikos Bay (northeastern Greece) by local fishermen in May 2021. Animals were transferred to the laboratory facilities, thoroughly washed under tap water, and placed in two aerated 2000‐l tanks. The tanks contained natural seawater and were equipped with a recirculating water system with mechanical, biological filters, and a UV lamp. The biofilters were fully mature and capable of complete nitrification. After 2 days, 560 clams were randomly distributed among eight 1000‐L tanks (supplied with the same recirculating system) as follows: 30, 40, 60, and 150 animals in duplicate. Clams were placed in the 1000‐l tanks within square permeable baskets (50 × 50 × 10 cm) filled with fine gravel (Figure 1) to allow them to bury themselves at their convenience.

A two‐week acclimation period followed, where water temperature was 17.8°C ± 0.49°C and clams were fed on two species of live microalgae (*Tisochrysis lutea*, CCAP 927/14 and *Tetraselmis* spp., Mediteranean strain) at a 1:1 dry weight ratio. The weekly quantity of the (dry) microalgae was 3% of the live weight of the clams. Fluorescent lamps provided artificial light 16 h a day. Feces and other waste settling at the bottom of the tanks were removed daily using aquarium siphons. Temperature (Hanna HI98129, HANNA Instruments Inc., Woonsocket, RI, USA) and dissolved oxygen (Hanna HI9142) were measured once daily through electronic devices. The nitrogen compounds, total ammonia nitrogen (TAN), nitrites (NO_2_^−^), and nitrates (NO_3_^−^) were also measured every day through saltwater test kits (Tetra, Melle, Germany). Depending on the clam load in each tank, 3%–15% of the water was exchanged weekly with fresh seawater to maintain consistent water quality across all tanks. For instance, the tanks containing 150 animals received a 5‐fold higher water exchange (15%) compared to the control tanks (3%).

The maximum density used in our recirculation aquaculture systems (RAS) was intentionally kept low, at 0.15 clams per liter of water (or 3 kg/m^3^), to minimize potential density‐related stress. This density is considerably lower than the optimal stocking densities reported for other bivalves in RAS, such as *Crassostrea gigas* larvae, which can reach up to 50 larvae/mL (de Oliveira Ramos et al. 2021). Although there are no published studies on optimal densities for adult bivalves in RAS, well‐designed systems for fish species such as *Salmo salar* , *Oncorhynchus mykiss* , and *Dicentrarchus labrax* can sustain densities above 40 kg/m^3^ (Mugwanya et al. 2022).

### 2.2 Trial Setup and Sampling Procedure

After the acclimation phase, the temperature of the six tanks containing 40, 60, and 150 clams was adjusted to 22.5°C, 24.5°C, and 26.5°C, respectively, while the two tanks containing 30 animals remained at 18°C (Table 1). A larger number of clams were maintained at higher temperatures to ensure sufficient alive individuals for gene transcription analysis and genotyping in the later stages of the experiment. The control temperature was chosen so as to match the average seawater temperature in northeastern Greece in May (SeaTemperature.org 2024). Electronic aquarium heaters (Digital Aquarium Heater DR‐9300, Boyu, Guangdong Province, China) were used, and the temperature was increased at a rate of 0.1°C per hour. Hour zero at each treatment was set at the time when the temperature of the tank reached the appointed value. Four samplings were conducted by picking three random animals on day 1 (hour 24), day 3, day 12, and day 25. During the experiment, the daily routine and feeding were exactly as in the acclimation period, except for the temperature and the dissolved oxygen measurements, which were conducted twice a day. The six sampled individuals from the different treatments at each sampling time were anatomized, and their mantle tissue was dissected with sterilized tools. Subsequently, tissues were transferred to 1.5 mL tubes, immediately flash‐frozen in liquid nitrogen, and stored at −80°C. Mortality was recorded every day. Individuals were considered dead when their shells gaped and they did not shut their valves after multiple mechanical stimuli.

**TABLE 1:** Mean water parameter values during the experimental period. Values are mean ± standard deviation.

Tank	Temperature (°C)	Dissolved oxygen (mg/L)	pH	TAN (mg/L)	NO_2_^−^ (mg/L)	NO_3_^−^ (mg/L)
1	17.8 ± 0.59	6.9 ± 0.42	8.28 ± 0.17	0–0.25	0–0.25	9.8 ± 2.8
2	18.1 ± 0.44	6.7 ± 0.26	8.25 ± 0.14	0–0.25	0–0.25	9 ± 1.7
3	22.4 ± 0.34	6.7 ± 0.35	8.23 ± 0.07	0–0.25	0–0.25	11.5 ± 2.5
4	22.5 ± 0.41	6.5 ± 0.52	8.23 ± 0.19	0–0.25	0–0.25	13.2 ± 3.1
5	24.4 ± 0.51	6.4 ± 0.4	8.22 ± 0.12	0–0.25	0–0.25	15.8 ± 3.5
6	24.6 ± 0.35	6.3 ± 0.31	8.19 ± 0.16	0–0.25	0–0.25	13.6 ± 4.1
7	26.6 ± 0.55	6.2 ± 0.54	8.14 ± 0.11	0–0.25	0–0.25	19.4 ± 4.4
8	26.7 ± 0.38	6.1 ± 0.45	8.12 ± 0.1	0–0.25	0–0.25	22.6 ± 5.3

### 2.3 Animals for Genotyping

Thermal stress resilient and susceptible *R. decussatus* were collected for the sequencing of three antioxidant genes. The objective was the discovery of polymorphisms in the coding regions potentially associated with higher tolerance to thermal stress. Twenty clams that survived the 26.5°C exposure until day 25 were gathered and labeled as the resilient ones. The susceptible animals were collected during the experimental trial. These were five animals that passed away at 24.5°C between the days 11–14 when the rate of the total mortalities at 24.5°C was low and fifteen clams that died between days 7 and 11 at 26.5°C (the day after the first heavy mortality episode and before the stabilization of the mortality rate at 26.5°C). The mantles of the resilient and susceptible clams were dissected with sterile tools and stored at −80°C.

### 2.4 RNA Extraction and cDNA Synthesis

Total RNA was extracted from the mantles of the sampled clams on days 1, 3, 12, and 25 for the gene expression analysis and from the resistant and susceptible animals. RNA extractions were made in 136 *R. decussatus* mantles (96 for gene expression and 40 for genotyping) with the use of the NucleoZOL reagent (Macherey‐Nagel, Düren, Germany), following the guidelines of the manufacturer. Briefly, 50 mg of mantle tissue was homogenized by hand through RNase‐free disposable pestles in 1.5 mL sterile tubes along with 500 μL NucleoZOL. RNA‐ase free water was added to the samples and samples were centrifuged. The supernatant was transferred to new sterilized tubes and isopropanol was added to precipitate the RNA. Samples were again centrifuged, the liquid phase was discarded, and two 75% ethanol washes were performed. The RNA pellet was resuspended in 100 μL RNA‐ase free water. RNA concentration and purity were assessed in a Quawell UV–Vis 5000 spectrophotometer (Quawell Technology, San Jose, CA, USA) and RNA samples were stored at −80°C until the reverse transcription step. When the samples for gene expression showed low quality as determined by the *A*_260_/*A*_280_ ratio, they were discarded. For the reverse transcription step, 500 ng (gene expression) or 1000 ng (genotyping) of total RNA were used in the reaction along with the PrimeScript RT Reagent Kit (Takara, Otsu, Japan). All steps in the reverse transcription were performed according to the kit protocol. cDNA was diluted and samples were stored at −20°C until PCR applications.

### 2.5 Primer Design and Gene Expression Analysis

The transcriptional patterns of six genes (Table 2) involved in the antioxidant defense, anti‐apoptotic processes, and metabolism were evaluated by quantitative real‐time PCR (qPCRs). The expression of each target gene was calculated relative to the mRNA levels of the same gene in the control treatment (18°C) within each sampling time. In total, 80 samples (5 clams/treatment/sampling time) were analyzed for each gene. PCR reactions were run in the Thermocycler Eco 48 Real‐time PCR (Illumina, San Diego, CA, USA) in 10 μL final volume. Each well contained 10 ng of cDNA as template, 5 μL of KAPA SYBR FAST qPCR Master Mix, 2 μΜ of each primer, and PCR‐grade water until 10 μL. The primers used are shown in Table 2. The efficiency of all primer pairs was calculated by running preliminary qPCRs in five‐fold dilutions of 5 random cDNA samples and through the equation *E* = 10^(−1/slope)^ (Pfaffl 2001). The slope was calculated from the regression line of the C_t_ values from the five serial five‐fold dilutions versus the relative concentration of cDNA.

**TABLE 2:** Primer sequences, efficiencies (*E*), annealing temperature, amplicon size, and target sequence accession number of the genes analyzed in quantitative PCR.

Gene	Forward primer (5′‐3′) Reverse primer (5′‐3′)	*E* (%)	Annealing	Amplicon (bp)	GenBank accession number	References
Cu/Zn superoxide dismutase	TGTGCATGAGTTTGGTGACA CACTCTCATCAGCTGTGACA	93.9	53°C	140	PQ072799	Papadopoulos, Giantsis, et al. (2025); Papadopoulos, Michaelidis, and Giantsis (2025)
Catalase	GAGCAGGGGCATTTGGATAC TCACCACCAACTGTAGAGAAC	97	56°C	124	PQ073188	This study
Metallothionein	GCTGCAAATGTGGACCAAAC GCATGAGCAATCACTACCACA	103.8	56°C	146	EU000310.1 PQ073187	This study
B‐celllymphoma 2	GAAGACGGTCAAATAAATTGG CCTCCATTATCTAAAATCCATC	96.1	50°C	167	KC506419.1 MH628669.1 KX279941.1	This study
Pyruvate kinase	GATCTCAAGTTTGGKGTKGA CTCTGGWGGAATTTCKATACC	98.3	50°C	231	AM076953.1 MH220522.1 XM_045328067.2	This study
Phosphoenolpyruvate carboxykinase	ATGCAYGACCCAATGGCAATG CGGAACCAGTTCACATGGAA	101.4	58°C	125	AM076953.1 XM_060739733.1	This study
18S ribosomal RNA	CCCATTCGTGCTAGGGATTG GTACAAAGGGCAGGGACGTA	98.8	54°C	106	KX713340.1	Papadopoulos, Giantsis, et al. (2025); Papadopoulos, Michaelidis, and Giantsis (2025)

All primers were designed based on selected sequences (Table 2, Table 3) through the *primer3* software (version 4.1.0.) (Rozen and Skaletsky 1999). For gene expression analysis, the prediction of primer dimers and hairpin formation was made in *Oligo Analyzer 1.0.2*. When the primer pair efficiency was not between 90% and 110%, a new primer pair was designed. Melt curves were constructed after all runs to verify the absence of double peaks and primer dimers. The threshold cycle (*C*_*t*_) values of target genes in each sample were normalized to the corresponding *C*_*t*_ values of the reference gene (18S ribosomal RNA) as it had a stable expression at the different temperatures and sampling times. Relative expression of all target genes was estimated using the comparative *C*_*t*_ (2^–ΔΔ*Ct*^) method (Livak and Schmittgen 2001), since all primer pair efficiencies did not differ more than 5% from the efficiency of the 18S rRNA primers (Table 2). The primers for the amplification of the genes *Cu/Zn‐sod*, *bcl2*, *catalase*, *pk*, and *pepck* were designed within conserved regions identified from multiple alignments of gene sequences from several related bivalve species (Tables 2, 3) since there were no available sequences for *R. decussatus* in public databases.

**TABLE 3:** Primers and annealing temperature for the amplification of the genes that were sequenced for polymorphisms.

Gene	Forward primer (5′‐3′) Reverse primer (5′‐3′)	Annealing	Expected amplicon size (bp)	GenBank accession number
Catalase	GAGAGGACCAATTCTCCTTGA CATCTTTCATGTGACCAGCAA	52°C	1274	KF673103.1 XM_060712534.1
Metallothionein	ATTGTGTTGAAACTGGAAGCT GTGGAAATTATGAAACCACCA	53°C	338	EU000310.1
Cu/Zn superoxide dismutase	TGTCACTTCTTGCAAAGTGTGTT TGGCAATTCCAATAACACCACA	52°C	456	JQ362416.1

### 2.6 Sequencing and Sequence Analysis of Resilient and Susceptible Clams

Three genes were successfully amplified in the resistant and susceptible clams (Table 3). PCR reactions were carried out in the thermo‐cycler FastGene ULTRA Cycler (NIPPON Genetics EUROPE, Düren, Germany). All samples were amplified using 10 μL of FastGene Taq 2 × Ready Mix (NIPPON Genetics EUROPE), 0.4 μΜ of each primer, 30 ng of the template cDNA, and PCR‐grade water up to the final volume (20 μL). The thermal cycling regime consisted of an initial denaturation step of 3 min at 95°C, followed by 38 cycles of denaturation (95°C for 30 s), primer annealing (50°C–53°C for 30 s, Table 3), and extension (72°C for 30–90 s, determined by the size of the target) and finally, a last extension step of 10 min at 72°C. PCR products were examined by electrophoresis on a 1.2% agarose gel stained with Midori Green Advance (NIPPON Genetics EUROPE) and visual observation of the results under UV light.

All accurately amplified PCR products were cleaned using the NucleoSpin Gel and PCR Clean‐up kit (Macherey Nagel, Düren, Germany). The purified samples were sequenced in both directions in an ABI 3730xl automatic sequencer. Sequencing quality was evaluated by visual examination of the derived chromatograms using Finch TV 1.4.0 (Geospiza, Seattle, WA, USA) and BioEdit (Hall 1999). The sequences were aligned in MEGA X (Kumar et al. 2018) with the help of the MUSCLE algorithm.

### 2.7 Annotation and Structure of the Proteins

The obtained DNA sequences were translated into amino acids in MEGA X. The derived amino acid sequences were uploaded to the I‐TASSER online server (Yang et al. 2015) to generate the three‐dimensional (3D) protein structures. The best‐supported structure, identified by the highest C‐score (Zhang 2008), was further processed using the molecular graphic software PyMOL (version 3.0.1) (https://www.pymol.org/pymol). Protein domain annotation, as well as identification of ligand binding sites and active sites, was based on I‐TASSER predictions and was also validated from available public databases annotations of homologous proteins from related bivalve species.

### 2.8 Statistical Analysis

Gene transcription was quantified relative to the control clams at each sampling time point. Prior to analysis, data were initially tested for normality by conducting the Shapiro–Wilk test and for homogeneity of variances using the Brown‐Forsythe test. Depending on the outcome of normality tests, data were analyzed using either one‐way analysis of variance (ANOVA) or the non‐parametric Kruskal–Wallis test, with a significance level set at *p* < 0.05. If significant differences among temperature treatments were detected by ANOVA, Tukey's HSD multiple post hoc comparisons were performed to define the groups that statistically differ (*p* < 0.05). For the Kruskal–Wallis test, multiple post hoc comparisons with Bonferroni correction were used to identify significantly different groups (*p* < 0.05). All statistical analyses and figures were made in GraphPad Prism (version 8.4.2) and results of relative gene expression were expressed as means ± standard deviation. Statistical analysis of nucleotide and genotype frequencies of the identified polymorphisms in resistant and susceptible clams was performed using the chi‐squared (*χ* ^2^) test (Table S1) at a significance level of α = 0.05 (95% confidence).

## 3 Results

### 3.1 Mortality

**FIGURE 2:**
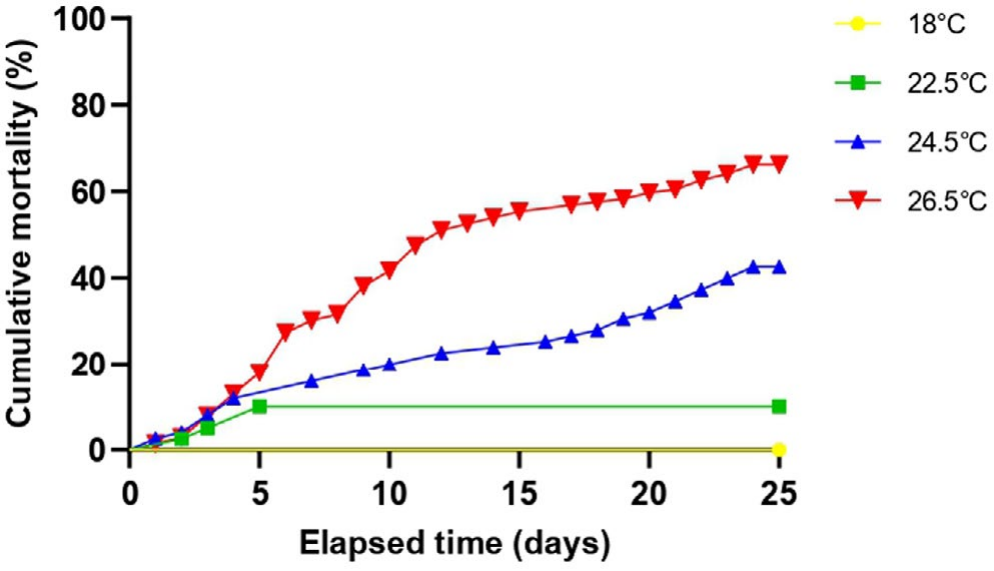
Cumulative mortality in the different treatments during the experiment.

All clams survived the control temperature (18°C) after 25 days. Αt 22.5°C mortality reached 10% on day 5, and no further death was recorded until day 25 (Figure 2). The death rate of clams exposed to 24.5°C was initially high and stabilized after day 4. The mortality of this group surpassed 20% on day 14, while the final mortality was 38.44% (Figure 2). At the highest tested temperature, the mortality rate was relatively high until day 12, when it reached 50%. Afterward, deaths at 26.5°C slowed and reached 66.19% on day 25.

### 3.2 Transcription of Antioxidant Genes

**FIGURE 3:**
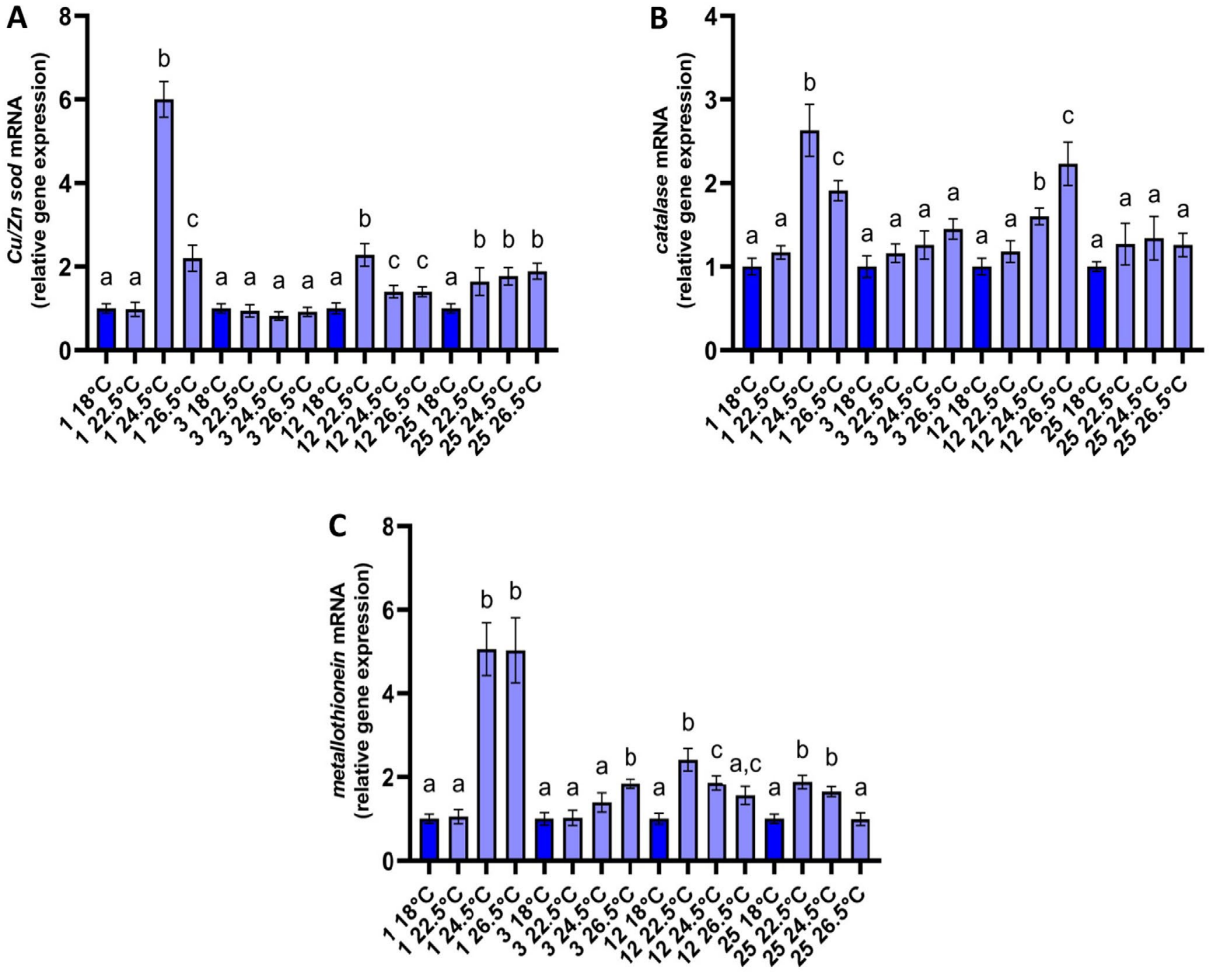
Relative mRNA levels of *Cu‐Zn sod* (A), *catalase* (B), and *metallothionein* (C) in the mantles of *Ruditapes decussatus*. Relative expression was calculated in relation to the control (18°C) within each sampling time. Values are means ± SD, *n* = 5 clams. Lowercase letters indicate significantly different means (*p* < 0.05) within a given sampling time. Dark blue depicts the control treatment.

The transcription of *Cu‐Zn sod* in the mantles of *R. decussatus* at 22.5°C was initially at the control levels. However, on days 12 and 25, the mRNA levels of this gene were significantly increased (Figure 3A). Clams at 24.5°C and 26.5°C had always significantly increased mRNA levels of *Cu‐Zn sod* compared to 18°C, except for day 3 when all treatments exhibited equal transcription (Figure 3A). Similar transcription of *Cu‐Zn sod* was observed at 24.5°C and 26.5° on days 12 and 25 (Figure 3A). *Catalase* mRNA at 22.5°C remained unchanged compared to control clams throughout the exposure (Figure 3B). In contrast, the exposure of clams at 24.5°C and 26.5°C led to significantly elevated *catalase* transcription on day 1 and day 12 (Figure 3B). The expression of *metallothionein* was found significantly increased at 22.5°C during the later stages of the exposure, on days 12 and 25 (Figure 3C). At 24.5°C, the clams displayed generally higher transcription of *metallothionein* throughout the experiment compared to 18°C, while at 26.5°C, the mRNA levels of this gene were only at the first days of exposure significantly elevated. Long‐term exposure at 26.5°C resulted in similar to 18°C transcription of *metallothionein* (Figure 3C).

### 3.3 Transcription of *bcl2*

**FIGURE 4:**
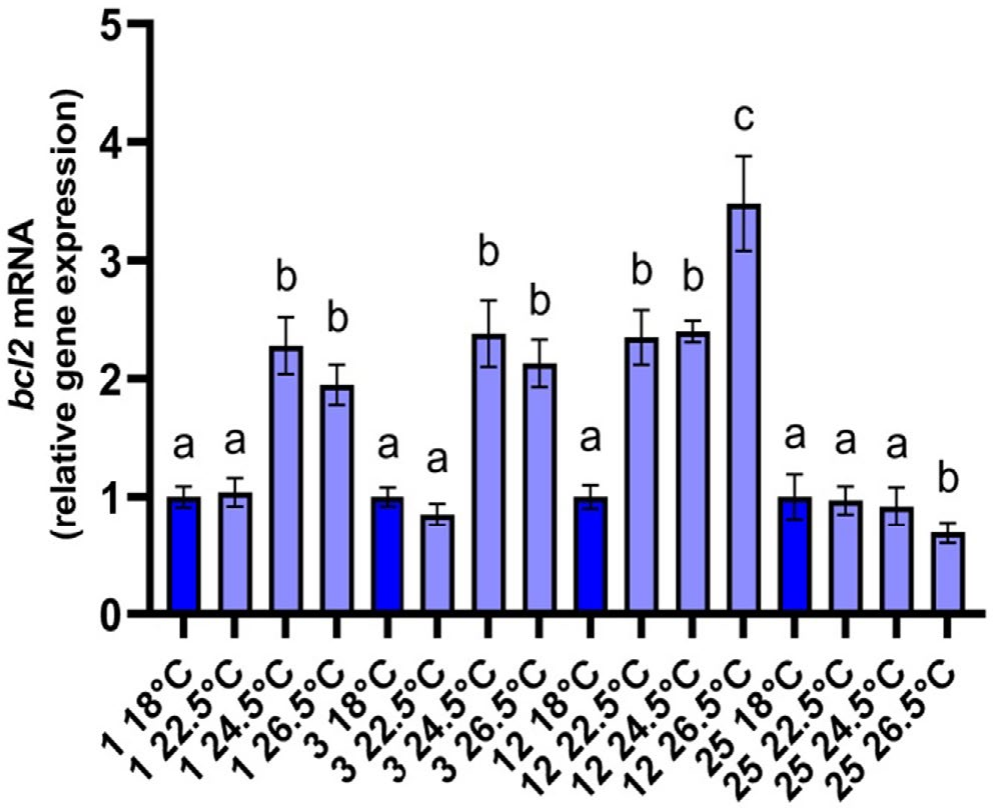
Relative mRNA levels of *bcl2* in the mantles of *Ruditapes decussatus*. Relative expression was calculated in relation to the control (18°C) within each sampling time. Values are means ± SD, *n* = 5 clams. Lowercase letters indicate significantly different means (*p* < 0.05) within a given sampling time. Dark blue depicts the control treatment.

The exposure of *R. decussatus* at 22.5°C led to the equivalent of 18°C transcription of *bcl2*, since the mRNA levels differed only on day 12, when they were significantly increased (Figure 4). The mRNA of *bcl2* in the mantles of clams at 24.5°C was raised significantly during the first 12 days, contrasted with the animals at 18°C, and on day 25, returned to the control levels (Figure 4). In parallel, significantly increased *bcl2* transcription was also found until day 12 in animals exposed to26.5°C. However, on day 25 the mRNA levels of *bcl2* in the mantles of these animals were significantly decreased compared to all other treatments (Figure 4).

### 3.4 Transcription of *pk* and *Pepck*

**FIGURE 5:**
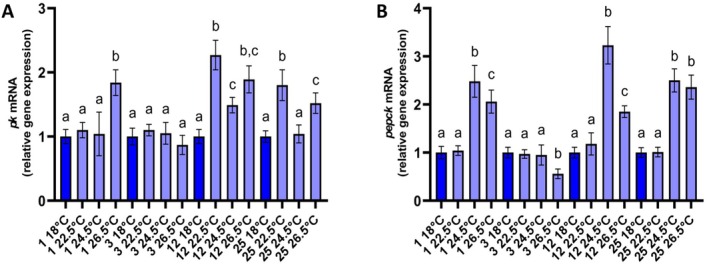
Relative mRNA levels of *pk* (A), and *pepck* (B) in the mantles of *Ruditapes decussatus*. Relative expression was calculated in relation to the control (18°C) within each sampling time. Values are means ± SD, *n* = 5 clams. Lowercase letters indicate significantly different means (*p* < 0.05) within a given sampling time. Dark blue depicts the control treatment.

The transcription of *pyruvate kinase* was stable among the treatments until day 3, with the exception of a statistically significant increase at 26.5°C on day 1 (Figure 5A). On day 12, all treatments exhibited significantly increased mRNA levels of *pk* compared to the control clams. On day 25, clams at 22.5°C and 26.5°C exhibited increased *pk* transcription, while those at 24.5°C had similar to control transcription (Figure 5A). The mRNA of *pepck* was significantly increased after 24 h at the highest tested temperatures, while it returned to the control levels by day 3 at 24.5°C (Figure 5B). In contrast, the animals at 26.5°C exhibited significantly reduced *pepck* transcription on day 3, compared to the other treatments. Later, on days 12 and 25, the mRNA levels of *pepck* were found to increase at 24.5°C and 26.5°C, while no significant change occurred in the levels of *pepck* at 22.5°C, in comparison with the control clams (Figure 5B).

### 3.5 Identified Polymorphisms and Their Distribution Among Resistant and Susceptible Clams

The genes *catalase, metallothionein*, and *Cu/Zn‐sod* were successfully amplified and sequenced. The identified polymorphisms include two non‐synonymous SNPs in *catalase* and *metallothionein* and a single synonymous SNP in *Cu/Zn‐sod* with significantly differential distribution among the resilient and the susceptible clams. The non‐synonymous substitution in the cds of *catalase* is located at position 1093 of the amplified sequence (GenBank Acc. No. PQ073188), and is caused by an A to T transversion that results in a substitution of Asn to Tyr at amino acid 422 of the corresponding signal peptide of the most similar complete sequence that was available in GenBank (*Paratapes textilis*, KF673103.1). The distribution of the genotypes A/A and T/T did not differ significantly in the two groups of clams. However, the nucleotide frequencies varied significantly among resilient and susceptible animals (Table 4) and thus the allele A was associated with increased susceptibility to thermal stress.

**TABLE 4:** Distribution of polymorphisms in the coding sequences of *catalase*, *metallothionein*, and *Cu‐Zn sod* among resistant and susceptible clams. The identified substitution in the partial 3′ untranslated region (3′ UTR) of *metallothionein* is also included.

Position	Genotypes	Susceptible	Resistant	Mutation type	Allele	Susceptible	Resistant
Catalase							
cds‐1093*	A/A	17 (94.4)	13 (72.2)	Asn (N) or Tyr (Y) AAT or TAT	A T	**34 (94.4)** **2 (5.6)**	**26 (72.2)** **10 (27.8)**
	T/T	1 (5.6)	5 (27.8)				
Metallothionein							
cds‐164	A/A	13 (76.4)	12 (75)	Gln (Q) or Pro (P) CAA or CCA	A C	28 (82.4) 6 (17.6)	28 (87.5) 4 (12.5)
	A/C	2 (11.8)	4 (25)				
	C/C	2 (11.8)	0 (0)				
3′ UTR‐56*	A/A	8 (47.1)	11 (68.8)	3′ UTR base substitution	A G	**20 (58.8)** **14 (41.2)**	**27 (84.4)** **5 (15.6)**
	A/G	4 (23.5)	5 (31.2)				
	G/G	5 (29.4)	0 (0)				
Cu‐Zn sod							
cds‐309*	C/C	9 (50)	14 (77.8)	Synonymous substitution ATC or ATT	C T	**20 (55.6)** **16 (44.4)**	**30 (83.3)** **6 (16.7)**
	C/T	2 (11.1)	2 (11.1)				
	T/T	7 (38.9)	2 (11.1)				

* Indicates significant differences between resistant and susceptible animals. Τhe specific differences are shown in bold.

The amplified sequence of *metallothionein* corresponds to the fifth amino acid onwards of the previously deposited sequence for *R. decussatus* (GenBank Acc. No. EU000310.1) and the corresponding peptide (GenBank Acc. No. ABS20116.1). In total, 129 base pairs of the 3′ untranslated region were also amplified. One non‐synonymous substitution was found in the coding region at cds‐164 of the deposited sequence (GenBank Acc. No. PQ073187). This was a dimorphism caused by an A to C transversion that changes the Gln into Pro at position 55 of the corresponding signal peptide (XDE91521). Genotype and nucleotide frequencies did not significantly vary among resistant and susceptible clams (Table 4). In the 3′ UTR, one SNP was identified and exhibited different nucleotide frequency among resistant and susceptible animals. However, this polymorphism (3′ UTR‐56) did not show significantly different genotype frequencies (Table 4). Therefore, the nucleotide G at position 56 of the 3′ UTR was linked to increased susceptibility to thermal stress.

The cds of the intracellular *Cu‐Zn sod* of *R. decussatus* was completely amplified and deposited in GenBank (Acc. No. PQ072799). The cds consists of 462 base pairs encoding a 153 amino acid peptide. This superoxide dismutase was predicted by I‐TASSER to be the cytosolic one (GO‐Score = 1) and thus this enzyme is the intracellular Cu‐Zn SOD of the species. Only synonymous substitutions were detected in the 462 base pairs of the cds. One of them (cds‐309) displayed significant differences in nucleotide but not in genotype frequencies in susceptible and resistant clams (Table 4). Thus the presence of T was linked to increased susceptibility to thermal stress.

### 3.6 Three‐Dimensional (3D) Structure, Annotation, and Visualization of the Signal Peptides

#### 3.6.1 Catalase

**FIGURE 6:**
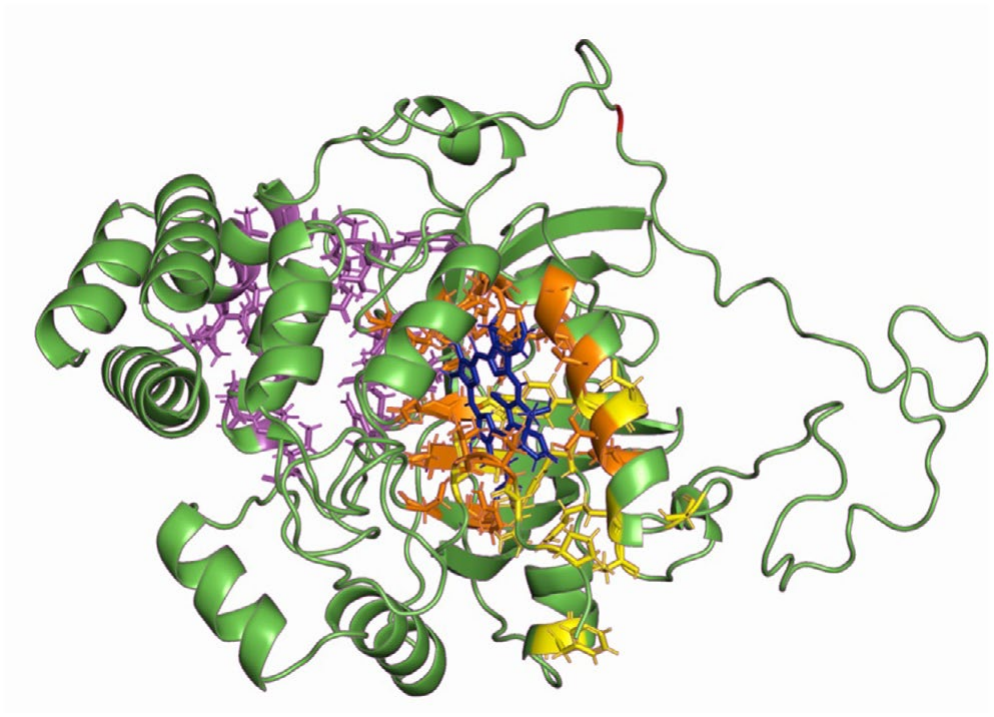
The three‐dimensional (3D) structure of the *Ruditapes decussatus* Catalase monomer (*C*‐score = 1.49). 65 and 17 amino acids are missing from the N‐terminal and C‐terminal ends respectively. The heme binding site is shown as orange‐colored sticks, and the NADPH binding site is highlighted as magenta‐colored sticks. Yellow sticks represent the active site (*C*‐score = 0.69). The amino acid that changes from the single identified polymorphism appears in red.

The partial cds region amplified from *catalase* corresponds to the amino acids 57–449 of the most similar complete deposited sequence (*Paratapes textilis*, KF673103.1, 90.1% similarity). The amplified region contains all the important regions of the enzyme. More specifically, the heme binding pocket was colored in orange sticks (*C*‐score = 0.92), the NADPH binding region is shown as magenta sticks (*C*‐score = 0.12), and the active site of the enzyme is depicted as yellow sticks (*C*‐score = 0.69) in Figure 6. Based on the protein annotation, the position of the amino acid residue affected by the mutation is not within any important region of the enzyme.

#### 3.6.2 Metallothionein

**FIGURE 7:**
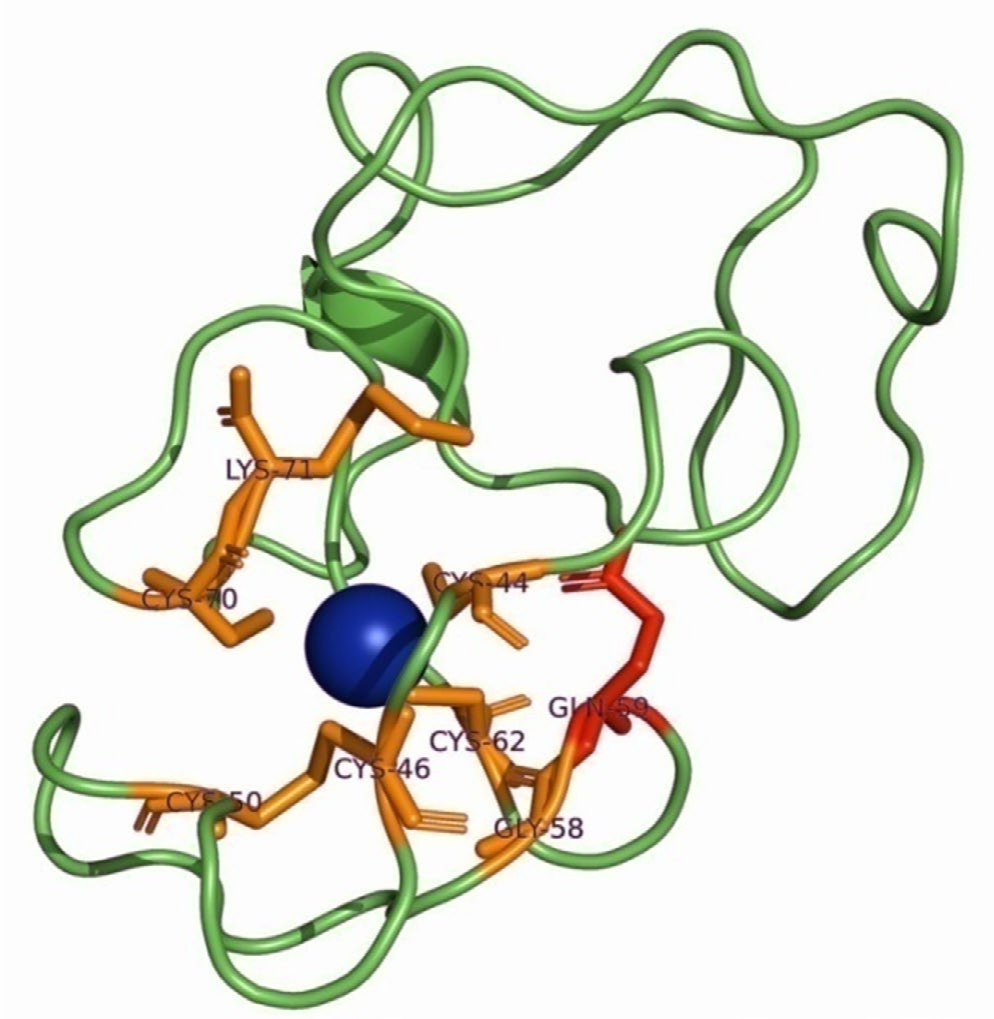
The three‐dimensional (3D) structure of *Ruditapes decussatus* Metallothionein (*C*‐score = −0.59). Cd was selected and its selection was expanded by 5 Å in PyMOL and the amino acids within this range were highlighted as orange sticks. The amino acid that changes from the detected polymorphism appears as red.

The three‐dimensional (3D) structure of Metallothionein is shown in Figure 7. All the 69 amino acids were included in the visualization of the peptide, even 65 were derived from sequencing, by obtaining the first 4 from the deposited signal peptide of *R. decussatus* (GenBank Acc. No. ABS20116.1). Cadmium (Cd) was selected in PyMOL, and the selection was expanded by 5 Å to identify the amino acids that are important for the Cd binding. These residues were depicted as orange sticks. The amino acid that changes due to the identified substitution (Gln‐59, red) is not among them, but it is adjacent to the Cd‐binding site (Figure 7).

#### 3.6.3 Cu‐Zn Sod

**FIGURE 8:**
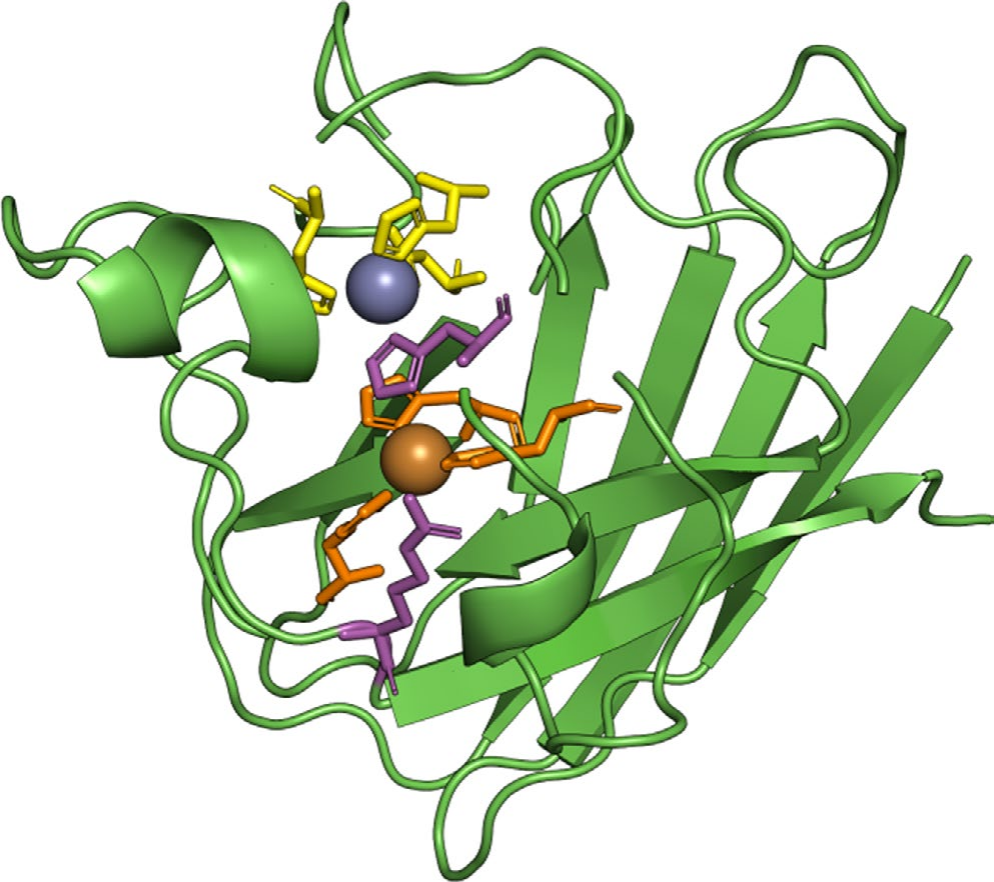
The three‐dimensional (3D) structure of *Ruditapes decussatus* Cu‐Zn SOD monomer (*C*‐score = 1.35). The amino acids of Cu and Zn binding sites are shown as sticks, colored in orange and yellow, respectively. The active site residues are highlighted as magenta‐colored sticks.

The residues of the Cu and Zn binding sites are depicted as orange sticks (*C*‐score = 0.95) and yellow sticks (*C*‐score = 0.54), respectively (Figure 8). Magenta colored sticks represent the active site of the enzyme (*C*‐score = 0.82).

## 4 Discussion

At 18°C, *R. decussatus* exhibited complete survival. In contrast, exposure to 22.5°C resulted in minor mortality, occurring only within the first few days following the temperature increase. Mortality increased significantly at higher temperatures, reaching 39.66% at 24.5°C and 66% at 26.5°C. No mortality was reported by Rato et al. (2022) in *R. decussatus* at 15°C–23°C after 5 days of exposure. Similarly, Macho et al. (2016) recorded no mortality of *R. decussatus* at sediment temperatures of 27°C and 32°C after 4 days of exposure, and Velez et al. (2017) found only 7% mortality at 25°C after 28 days. In contrast, in the present study, clams exhibited 16.9% and 17.1% mortality at 24.5°C and 26.5°C, respectively, until day four, with cumulative mortality at 24.5°C reaching 40% after 25 days of exposure. This discrepancy may be attributed to differential environmental conditions of the collection sites. For example, Macho et al. (2016) studied clams in intertidal habitats where sediment temperatures can reach 30°C–31°C due to tidal exposure, whereas the clams used in our study originated from coastal habitats without tidal influence, and thus are likely less adapted to such thermal extremes. Additionally, a marked genetic subdivision was observed across Eastern and Western Mediterranean populations of *R. decussatus* (Cordero et al. 2014; Papadopoulos et al. 2024). Thus, differences in mortality rates are expected when populations are naturally exposed to distinct thermal environments, as the tolerance of aquatic organisms to temperature fluctuations is affected by the thermal conditions they typically experience (Sorte et al. 2011) and the thermal tolerance range determined for one location may only be accurate within a specific geographic area (Wilson 1981), especially when significant genetic differences are also present.

*R. phillipinarum* was found to employ pervasive valve closure, which is a less demanding strategy to deal with short‐term environmental stress instead of promoting energetically expensive cellular responses (Anacleto et al. 2014). However, this behavioral strategy is only short‐term efficient considering that it involves metabolic arrest and possible induction of anaerobic pathways, which may not be favorable for long periods (Falfushynska et al. 2020). In our work, clams at 22.5°C might have followed a relevant strategy at the initial stages of exposure since their antioxidant mechanisms and the transcription of *bcl2* were up‐regulated only after 12 days. Early in the exposure at 22.5°C, *pepck* and *pk* transcription remained unchanged. This suggests that the clams initially maintained similar glycolytic and gluconeogenic capacities to the control group. However, prolonged exposure at 22.5°C led to a higher aerobic glycolysis, likely as a compensatory mechanism to meet elevated energy demands, and to an enhancement of antioxidant and anti‐apoptotic gene transcription. Regarding the antioxidant response, our findings align with Velez et al. (2017), who observed increased activity of antioxidant enzymes in the gills of *R. decussatus* after 28 days at 21°C compared to 17°C. Nevertheless, the transcription of *catalase* in clams at 22.5°C did not exceed that of the control clams throughout the experiment. This is consistent with previous studies (Crespo et al. 2021; De Marchi et al. 2020), which reported decreased or unchanged catalase activity in *R. decussatus* exposed to mildly elevated temperatures.

The exposure of clams at 24.5°C and 26.5°C stimulated an increased transcription of all antioxidant genes after 24 h, and a return to the basal levels on day 3. Banni et al. (2014) also observed an initially increased transcription of *catalase* and *sod*, and a subsequent drop to the control levels on day 3, in *M. galloprovincialis* when subjected to increased temperatures. In addition, *R. decussatus* subjected to 24.5°C and 26.5°C rapidly increased the transcription of *bcl2*. These findings indicate a robust initial stress response, likely triggered by the intense and sudden temperature increase, which ultimately led to the equally high mortality rates at these temperatures until day four. Subsequently, as also observed at 22.5°C, clams appear to employ a restricted energy production strategy and a mild antioxidant response, accompanied by a sustained highly transcribed *bcl2* as the duration of thermal stress prolongs. This suggests a switch from an immediate, powerful response to a more measured approach for coping with ongoing thermal stress.

The excessive ROS within the cells combined with decreased activity of antioxidant defense indices may result in oxidative stress and apoptotic signal induction. In our study, prolonged exposure to 24.5°C and 26.5°C resulted in elevated transcription of the antioxidant and anti‐apoptotic genes by day 12, in combination with an increased aerobic capacity, particularly at 26.5°C. This shift may have contributed to the observed stabilization of the mortality rate from day 12 onwards at both temperatures. However, the increased transcription of *pepck* from day 12 indicates metabolic stress, possibly because metabolic demands over time surpassed aerobic capacity and triggered partial activation of anaerobic pathways. Georgoulis et al. (2024) also observed a delayed up‐regulation of *pepck* transcription after mild heat stress in *Ostrea edulis* . Therefore, under exposure to temperatures above 24°C, clams likely attempted to extend their passive thermal tolerance by enhancing anaerobic metabolic capacity and suppressing overall metabolism (Pörtner et al. 2017). However, metabolic arrest is not sustainable over extended periods, as prolonged hypoxia poses a threat to cellular homeostasis (Falfushynska et al. 2020), since the switch from aerobiosis to anaerobiosis may reduce ATP production efficiency (Sokolova et al. 2012).

On day 25, clams at 26.5°C exhibited again elevated aerobic glycolysis and increased *pepck* transcription. In addition, only *Cu‐Zn sod* was up‐regulated among the antioxidant genes, and *bcl2* levels significantly decreased, indicating that the extent of thermal stress may have activated apoptotic processes. In contrast, clams at 24.5°C on day 25 showed a reduction of *pk* mRNA at the control levels, a basal transcription of *bcl2*, and antioxidant capacity similar to or greater than the clams at 26.5°C. The adjustments observed at 24.5°C on day 25 possibly contributed to the almost zero mortality during the final 3 days of exposure, along with their pronounced reliance on anaerobic energy production. These findings are consistent with findings by Anestis et al. (2007) who reported that chronic exposure of *M. galloprovincialis* at temperatures above 24°C led to a decreased ratio of enzymatic activities PK/PEPCK, indicating the activation of anaerobic pathways due to the magnitude of thermal stress, even at moderately increased temperatures.

Interestingly, the late antioxidant response of clams at 26.5°C included the transcription of *metallothionein* at the control levels on days 12 and 25. As observed in our work, the mRNA levels of MTs were found to increase early (within 24 h) after exposure to rising temperatures in *M. galloprovincialis* and to decrease after 3 days (Banni et al. 2014). However, the MTs protein content increased within 24 h and remained elevated after 3 days, contrasting the mRNA patterns (Banni et al. 2014). Thus, the transcriptional profile of MTs may not reflect their levels during the response. The SNP identified in the 3’ UTR region of *metallothionein*, showed significantly different nucleotide frequencies among resilient and susceptible clams. Such SNPs may not affect the amino acid composition of the corresponding peptide, but can influence the stability of mRNA and its translational efficiency affecting the amount of the produced protein (Barreau et al. 2005; Mayr 2017). Given the crucial role of metallothioneins (MTs) in thermal stress response of various bivalve species (Banni et al. 2014; Ivanina et al. 2009; Piano et al. 2004), SNPs in such genes likely play a significant role in thermal resilience. In the scallop *Argopecten irradians* , a SNP in the promoter region of Metallothionein‐1 has been linked to increased heat tolerance (Yang et al. 2013). Finally, the single identified non‐synonymous SNP of the *metallothionein* gene exhibited no differences in nucleotide and genotype frequencies, but it may deserve further investigation since the mutation affects an amino acid adjacent to the metal‐binding site.

Natural selection plays a crucial role in preserving non‐synonymous mutations that enhance a species' adaptability to environmental factors (Zhou et al. 2023). Specific non‐synonymous SNPs in antioxidant genes may be the result of divergent adaptations to the fluctuating marine environment. For instance, Le Luyer et al. (2022) identified a non‐synonymous substitution in the SOD gene of *Pinctada margaritifera* , with differing frequencies and gene expression levels between two populations originating from environments with distinct thermal conditions. Furthermore, Bao et al. (2010) associated a non‐synonymous substitution in the coding sequence of Cu‐Zn SOD in *Argopecten irradians* , with its resistance to *Vibrio anguillarum* infections. A non‐synonymous substitution was identified in the *catalase* gene of *R. decussatus*, showing different nucleotide frequencies but similar genotype frequencies between resilient and susceptible clams. The affected amino acid was located at a position that is possibly not directly important for the activity and functionality of the enzyme according to the annotations of the signal peptide. Nonetheless, such amino acid substitutions can still significantly impact the structure, stability, interactions, and function of the protein (Alberts et al. 2002; Dobson 2003).

The comparisons of the intracellular *Cu‐Zn sod* gene sequences revealed one synonymous substitution with significantly differential nucleotide distribution. Accordingly, Papadopoulos, Giantsis, et al. (2025); Papadopoulos, Michaelidis, and Giantsis (2025) identified a synonymous SNP in the corresponding SOD gene of the Mediterranean mussel, which was correlated with increased resilience to thermal stress. Synonymous substitutions were traditionally considered “silent” and believed to not affect phenotype. However, it is now recognized that they can significantly impact transcription, translation, and mRNA stability through various mechanisms (Liu 2020; Nakahigashi et al. 2014). Synonymous mutations at certain genetic loci have been shown to affect fitness and contribute to adaptive responses (Lebeuf‐Taylor et al. 2019), while a recent study correlated a synonymous substitution with the upper thermal limit of the mussel *Mytilisepta virgata* (Tan et al. 2023).

## 5 Conclusion

Two decades ago, it was recognized that the estimated global warming could influence the performance and survival of marine organisms, since most of them already live close to their thermal tolerance limits (Helmuth et al. 2006; Hoegh‐Guldberg et al. 2007). Understanding how organisms respond to warmer environments is therefore essential for revealing their molecular and phenotypic adaptive potential in the face of climate change. Behavioral and physiological adaptations to the same stressor appear to vary among geographically distant populations and may be influenced by underlying adaptive genetic variation (Li et al. 2018). In this study, clam mortality differed markedly from previous reports on *R. decussatus* from the Western Mediterranean and European Atlantic coasts. Clams from the northeastern Mediterranean were found to be highly vulnerable to sustained temperatures above 24°C, experiencing substantial mortality. This sensitivity may be partially attributed to the relatively rapid temperature increases used in our experimental design, which likely induced acute stress responses, in contrast to the more gradual thermal changes occurring in nature that may permit acclimatization. Furthermore, significant genetic differentiation has been documented between Eastern Mediterranean and Western Mediterranean/European Atlantic populations of *R. decussatus* (Cordero et al. 2014; Papadopoulos et al. 2024), which could underlie population‐specific thermal tolerance and contribute to the observed differences in mortality.

Following the first day of exposure, clams exhibited a conservative response. Since this strategy is not favorable for extended periods, anaerobic metabolism was activated at temperatures above 24°C, while at 26.5°C apoptosis was likely activated based on the down‐regulation of *bcl2* transcription. This study focused on the transcriptional regulation of six key stress‐responsive genes in the mantle tissue of the clams. Mantle tissue was selected due to its critical role in energy storage, metabolic activity, and overall physiological regulation in bivalves (Gosling 2015). However, future studies should also investigate other tissues, such as gills or muscle, incorporating protein level analyses and more advanced techniques, such as transcriptomics and proteomics, to complement the observed compensatory adjustments and offer a more comprehensive understanding of thermal responses in *R. decussatus*.

Eventually, three SNPs in *catalase*, *metallothionein*, and *Cu‐Zn sod* were associated with thermal stress resilience, representing potential markers for selective breeding of this clam. Although the study focused on a single population from the northeastern Mediterranean, previous studies have demonstrated limited genetic differentiation among *R. decussatus* populations within this region (Papadopoulos et al. 2024), suggesting that our findings may be broadly applicable to the Eastern Mediterranean. However, the genetic subdivision between Eastern and Western Mediterranean/European Atlantic populations of *R. decussatus* (Cordero et al. 2014; Papadopoulos et al. 2024) underscores the importance of caution when generalizing such results beyond the study area, while further research involving multiple populations is necessary to validate the relationship between these polymorphisms and thermal stress resilience across the species' range. Moreover, the small number of individuals genotyped for polymorphisms, combined with the weak correlations observed in several cases, emphasizes the need for further research to confirm the relationship between these polymorphisms and thermal stress resilience. Selective breeding may contribute to more robust and resilient stocks of *R. decussatus*, finally enhancing aquaculture practices and improving the sustainability of wild populations, especially in the context of global warming. Previously, aquaculture breeding goals primarily focused on traits like growth rate and disease resistance. However, heat tolerance is expected to become a major objective in future selective breeding programs. The newly characterized SNPs identified in this study may be particularly important toward this scope.

## Author Contributions

**Dimitrios K. Papadopoulos:** conceptualization (equal), methodology (lead), software (lead), validation (equal), writing – original draft (lead). **Basile Michaelidis:** conceptualization (equal), resources (lead), visualization (lead). **Ioannis A. Giantsis:** methodology (supporting), project administration (lead), supervision (lead), writing – review and editing (lead).

## Conflicts of Interest

The authors declare no conflicts of interest.

## Supporting information


**TABLE S1.** The chi‐squared test results for the distribution of genotypes and nucleotides in the sequences of *catalase, metallothionein*, and *Cu/Zn‐sod* among resilient and susceptible *R. decussatus*. Asterisk (*) indicates statistically significant differences.

## Data Availability

The amplified sequences of *Cu‐Zn sod*, *metallothionein*, and *catalase* have been deposited in the National Center for Biotechnology Information repository (NCBI) with the accession numbers PQ072799, PQ073187, and PQ073188, respectively. https://www.ncbi.nlm.nih.gov/nuccore/PQ072799; https://www.ncbi.nlm.nih.gov/nuccore/PQ073187; https://www.ncbi.nlm.nih.gov/nuccore/PQ073188.
