# Supplementary material for: Heat Stress Response in Ruditapes Decussatus: Transcriptional Regulation of Key Pathways and Novel SNPs in Antioxidant Genes
Source: Ecol Evol. 2025 Jun 10;15(6):e71563. doi: 10.1002/ece3.71563 (PMC12151883; doi:10.1002/ece3.71563)
Supplement: Supplementary file 1 — TABLE S1. The chi‐squared test results for the distribution of genotypes and nucleotides in the sequences of catalase, metallothionein, and Cu/Zn‐sod among resilient and susceptible R. decussatus. Asterisk (*) indicates statistically significant differences. [file ECE3-15-e71563-s001.docx]

| **Position** | **Genotypes** | **χ^2^** | **p** | **Nucleotide type** | **χ^2^** | **p** |
| --- | --- | --- | --- | --- | --- | --- |
| ***catalase*** | | | | | | |
| cds-1093 | A/A | 3.2 | 0.073 | A/T | 6.4 | 0.011* |
|  | T/T |  |  |  |  |  |
| ***metallothionein*** | | | | | | |
| cds-164 | A/A | 2.68 | 0.262 | A/C | 0.34 | 0.559 |
|  | A/C |  |  |  |  |  |
|  | C/C |  |  |  |  |  |
|  |  |  |  |  |  |  |
|  |  |  |  |  |  |  |
|  |  |  |  |  |  |  |
|  |  |  |  |  |  |  |
|  |  |  |  |  |  |  |
|  |  |  |  |  |  |  |
| 3’ UTR-56 | A/A | 5.56 | 0.062 | A/G | 5.25 | 0.021* |
|  | A/G |  |  |  |  |  |
|  | G/G |  |  |  |  |  |
| ***Cu-Zn sod*** | | | | | | |
| cds-309 | C/C | 3.86 | 0.144 | C/T | 6.55 | 0.011* |
|  | C/T |  |  |  |  |  |
|  | T/T |  |  |  |  |  |

**Heat stress response in *Ruditapes decussatus*: Transcriptional regulation of key pathways and novel SNPs in antioxidant genes**

Dimitrios K. Papadopoulos, Basile Michaelidis, Ioannis A. Giantsis

**SUPPLEMENTARY TABLE 1** The chi-squared test results for the distribution of genotypes and nucleotides in the sequences of *catalase, metallothionein*, and *Cu/Zn-sod* among resilient and susceptible *R. decussatus*. Asterisk (*) indicates statistically significant differences.
